# Artificial intelligence development and dissemination impact on the sports industry labor market

**DOI:** 10.3389/fspor.2024.1363892

**Published:** 2024-03-28

**Authors:** Ekaterina Glebova, Dag Øivind Madsen, Paulína Mihaľová, Gábor Géczi, Alexandra Mittelman, Bojan Jorgič

**Affiliations:** ^1^ Université Paris-Saclay CIAMS, Orsay, France; ^2^ USN School of Business, University of South-Eastern Norway, Hønefoss, Norway; ^3^ Faculty of Management, Comenius University Bratislava, Bratislava, Slovakia; ^4^ Department of Sport Management, Hungarian University of Sports Sciences (HUSS), Budapest, Hungary

**Keywords:** AI, artificial intelligence, sports, labour market, technological transformation, employment & career, workforce, job role

## Abstract

**Purpose:**

The objective of this study is to explore the impact of artificial intelligence (AI) development on the sports industry labor market, the ways in which AI has influenced the demand for labor, created new job opportunities, and impacted existing job roles.

**Methodology:**

It refers to the inductive approach in the spirit technological determinism theory. It is based on the literature review and written qualitative, semi-structured interviews (*N* = 14) with sports human resources, management, and technology professionals (purposive sampling). Analysis involved inductive coding and line-by-line analytics of the data.

**Findings:**

The labor market implications of AI in the sports industry are multifaceted. New job roles are likely to emerge, demanding a blend of AI expertise, data-analysis skills, and sports domain knowledge. Professionals in roles such as sports data analysts and marketing experts may find increasing opportunities. However, certain jobs undergo transformation as AI automates routine tasks. It requires individuals to upskill or transition to roles that require a deeper understanding of AI. This necessitates the creation of responsibilities focused on ethical AI governance and oversight.

**Originality:**

It is important to research the impact of AI dissemination on the sports industry labor market in a holistic manner because the effects of AI are complex and far-reaching. While there are potential benefits to the implementation of AI, there are also potential risks and challenges that need to be addressed, the implementation of AI in the sports industry could have broader social and ethical implications that need to be considered.

## 1 Introduction

The emergence and widespread adoption of artificial intelligence (AI) in the sports industry have already caused significant shifts in the labor market (1, 2). Furthermore, scholars predict a growing trend of labor market transformation due to AI development and wide dissemination (3). AI technology has enabled the gathering, analysis, and interpretation of copious amounts of data from diverse sources in the sports industry (4). This includes data on sport training and performance, sports journalism (5) and content creation, fan engagement, and ticket sales, among others. AI algorithms can analyze this data and provide insights that can assist coaches and sports organizations in making data-driven decisions (6). As a result, the demand for professionals who can analyze and interpret this data has risen, creating new job opportunities. As for the performance-tracking, AI-powered sensors can track athletes' movements and provide real-time feedback on their performance (7), allowing coaches to tailor their training and game strategies (8). This technology has resulted in new jobs for engineers, data scientists, and software developers who can design and maintain these systems. AI technology has also enhanced the fan experience by providing personalized recommendations for content and merchandise and creating new types of experiences that enrich the spectator experience (9, 10). This has led to the creation of new jobs in marketing, sales, and digital media.

Jobs displacement and labor market transformation can be seen as a result of AI development and diffusion (3). While AI has created new job opportunities (1), it has also led to the displacement of some jobs in the sports industry (4). A number of tasks that were once performed manually, such as data entry, can now be automated using AI, for example, ticket sales from a physical ticket office and “manual” control at the stadium entrance has been replaced by online digital ticketing and stadium access with QR codes (11). As a result, some lower-skilled jobs in the industry may be replaced by automated systems. The impact of AI on the sports industry labor market is multifaceted, and it is likely that we will continue to witness new job opportunities being created while some jobs become obsolete. However, the widespread adoption of AI technology in the sports industry is likely to raise the demand for highly skilled professionals who can work with these systems (6).

It is important to research the impact of AI development and dissemination on the sports industry labor market in a holistic manner because the effects of AI are complex and far-reaching. While there are potential benefits to the implementation of AI technologies in the sports industry, there are also potential risks and challenges that need to be addressed. Notably, AI could potentially lead to job displacement in some areas of the industry, particularly for less experienced or entry-level positions. This could have a significant impact on the labor market in the sports industry and require a shift in the types of skills and expertise that are valued in the industry (4). Furthermore, the implementation of AI in the sports industry could have broader social and ethical implications that need to be considered (1). Namely, it concerns about privacy and data protection, singularity, human vulnerability, as well as issues the potential for AI to perpetuate existing biases and inequalities (6).

By taking a holistic approach to research, it is possible to better understand the potential impacts of AI on the sports industry labor market and develop strategies to address these impacts. This can help ensure that the benefits of AI are maximized while also minimizing potential risks and challenges.

The objective of this study is to explore the impact of artificial intelligence (AI) development and dissemination on the sports industry labor market through the prism of technological determinism theory. Thus, it aims to investigate the ways in which AI has influenced the demand for labor, created new job opportunities, and impacted existing job roles. Research questions:

1. How has the development and dissemination of AI technology affected the sports industry labor market in terms of job demand and job roles?
2. What new job opportunities have been created in the sports industry as a result of AI development and dissemination?
3. What are the skills and knowledge required for the new job roles created by AI technology in the sports industry?
4. In what ways has AI technology displaced existing job roles in the sports industry, and what are the consequences of this displacement?
5. What are trends, the potential future developments of AI in the sports industry, and what impact might these developments have on the labor market?

These research questions are be addressed through qualitative interviews with industry experts who possess knowledge and experience in the sports industry labor market and AI technology. The study aims to provide profound qualitative insights into the labor market dynamics and the changing nature of work in the sports industry, with the goal of contributing to the broader understanding of the impact of AI on labor markets and sports industry ecosystem (6).

## 2 Theoretical background

### 2.1 AI, technological determinism, and nowadays labor market landscape, AI types and applications

In general, AI is the simulation of human intelligence processes by machines, opposed to human and animal intelligence. There are different types of AI in use today, and there is often overlap between them, for example reactive machines, deep learning, natural language processing, and robotics, among others. Often AI systems combine multiple types of AI to function.

### 2.2 Technological determinism

Technological determinism is a theoretical perspective that posits technology as a primary driver (12) in shaping societal and cultural norms (13), rather than being shaped by them (14). Campolo and Crawford (15) introduce “enchanted determinism”, explaining it as a phenomenon of “superhuman” accuracy and insight, paired with the inability to clearly understand and explain how these results are produced. In the context of researching the impact of AI on the labor market in the sports industry, in this paper, the theoretical framework of technological determinism is applied to investigate the extent to which AI is one of driving forces in shaping labor market and sports ecosystem in general.

In applying the theoretical lens of technological determinism to this research topic, a thorough analysis of the current and future applications of AI within the sports industry are conducted. It emphasizes the impact of AI on the industry's workforce, such as changes in job demand, requirements and the emergence of roles.

### 2.3 Effect of AI on the labor market in general

The influence of AI on the labor market is a complex and multifaceted issue that requires a comprehensive perspective and holistic view. The introduction of AI has the potential to generate new employment opportunities, increase productivity, and enhance efficiency across various industries (16). However, it can also cause job displacement by automating tasks previously executed by human workers, potentially resulting in a rise in unemployment, stagnant wages, and an increase in income inequality. The impact of AI on the labor market is already being felt in certain sectors, such as manufacturing, services, transportation. There is an increasing usage of robots and other automated systems for tasks that were once done by human labor, including assembly line work or driving. While such developments have resulted in an increase in productivity and efficiency, they have also led to job losses and the displacement of human workers functions (both, physical and intellectual) by machines.

Nevertheless, in other industries such as healthcare and education, the impact of AI is less clear nowadays. AI has the potential to streamline administrative tasks and improve patient outcomes in healthcare, but it may not replace the human element of care that is essential to patients. Similarly, while AI can provide personalized learning experiences in education, it currently may not replace the crucial role of teachers and trainers in inspiring and guiding students.

In general, the impact of AI on the labor market is anticipated to be mixed, with some sectors and job categories being more vulnerable to changes than others (17). It is vital to take a holistic view of the issue, considering not only the potential benefits and drawbacks of AI but also the social and economic factors that shape the labor market. This encompasses issues such as income inequality, access to education and training, and the role of government in regulating the use of AI in the workplace.

### 2.4 AI applications in sports industry and future perspectives

#### 2.4.1 Current widespread applications of AI in sports

Today, AI can analyze vast amounts of data from sensors, video footage, and other sources to provide insights into player performance and game strategies (2). This can include analyzing technical aspects of play such as speed, endurance, and accuracy, as well as tactical and strategic considerations such as player positioning and game scenarios (6). AI can also be used to provide customized training regimens for individual athletes, identifying areas for improvement and tailoring training sessions to address specific weaknesses or disadvantages (18). Furthermore, AI-powered wearables and other devices can help coaches and trainers monitor athlete health and well-being, including tracking heart rate, sleep patterns, and other biometric data to optimize training regimens and reduce the risk of injury (4). These technologies can also provide real-time alerts to medical staff if a player shows signs of fatigue or injury.

AI can be used to improve the fan experience, including chatbots and virtual assistants that provide personalized and interactive experiences for fans (16). This can include answering questions, providing real-time updates, and assisting with ticketing and merchandise purchases. AI-powered voice assistants can also be used to create more immersive experiences, allowing fans to engage with sports content in new and exciting ways. Also, AI can help optimize scheduling, logistics, and other operational aspects of sports events, including parking, concessions, and security (11). By analyzing data on historical attendance and other factors, AI can help venue managers optimize staffing levels, queue management, and other key aspects of the customer experience and CRM.

In marketing, AI is able to analyze customer data and develop targeted campaigns (6) that resonate with sports fans. By analyzing factors such as demographic data, social media activity, and past purchase behavior, AI helps sports organizations tailor their messaging to specific audiences, increasing engagement and revenue (9, 11). AI is helpful in sports betting by analyzing data and making predictions about game outcomes and player performance. By combining vast amounts of data from a range of sources, including social media, weather forecasts, and historical performance data, AI algorithms can provide insights that help sports bettors make more informed decisions (2). AI can be used to identify and recruit talented athletes, particularly in less developed sports or regions. By analyzing performance data, video footage, and other factors, AI can help identify promising athletes who may otherwise go unnoticed. AI can provide assistance to referees in making decisions on game scenarios. By analyzing game footage (11), AI algorithms can provide real-time insights and even flag potential fouls or rule violations that may have been missed by human referees (4).

To this end, AI is rapidly transforming the sports industry, with a range of potential applications that can improve performance, judging, and optimize various aspects of sports marketing and management (1, 19). As the technology continues to evolve, innovative applications of AI in sports are expected in the future (2, 16).

#### 2.4.2 Perspective trends of AI in sports

Discussing the current state of AI applications, we should take into account that the development and dissemination of AI technology advances (2). There are several potential future trends that could have a significant impact on the sports industry: (1) predictive analytics (6), (2) Immersive technologies (XR) Virtual (VR) and augmented reality (AR) (20), (3) automated scouting and talent identification (4), (4) AI-powered medical diagnosis and treatment, (5) AI-powered sports journalism (5), (6) machine learning for personalization (9).

(1) AI-powered predictive analytics could provide insights to help teams and coaches make informed decisions about player recruitment, game strategies, and training regimens. By analyzing vast amounts of data on player performance, weather conditions, and other factors, predictive analytics could provide insights that help teams gain a competitive edge. (2) As XR technologies become more advanced, they could offer new opportunities for fan engagement and athelts training. For example, VR could be used to create immersive fan experiences that allow viewers to feel like they are part of the action. AR could be used to provide real-time insights and feedback to athlets, improving their performance on the field (6, 9). (3) AI could be used to automate the scouting and talent identification process, particularly in less developed sports or regions. By analyzing performance data, video footage, and other factors (4), AI algorithms could help identify promising athletes and bring them to the attention of scouts and coaches. (4) AI could be used to analyze player biometric data and provide real-time feedback (4) to coaches and medical staff (6). This could help prevent injuries and optimize training regimens, potentially extending players' careers and improving their performance. (5) AI could be used to analyze data on player performance, game outcomes, and other factors to generate real-time sports journalism content (5). This could help journalists and broadcasters provide more in-depth and informative coverage of sports events, and could even be used to generate automated game reports and social media content. (6) AI-powered machine learning could help teams and brands personalize their offerings to individual fans (16). By analyzing data on past purchase behavior, social media activity, and other factors, machine learning algorithms could generate customized recommendations for products and services that resonate with individual fans.

These trends show the potential for AI to revolutionize the sports industry in the future. However, there are also potential challenges and risks that need to be considered, such as the ethical implications of AI applications in sports, the potential for job displacement in certain sectors, and the need for appropriate regulations to ensure fair play. Overall, the development and dissemination of AI in the sports industry offers both opportunities and challenges that require careful consideration and management (1, 19).

#### 2.4.3 AI affected sectors of sports industry

AI is becoming increasingly prevalent in the sports industry (4), offering new possibilities for improving performance, enhancing the fan experience, and optimizing various aspects of sports management. Here are some of the current applications of AI in sports, including performance analysis (6), athlete monitoring, customer relationship management (CRM) and fan engagement (9), event management, marketing and advertising, sport journalistic, sports betting, talent identification (21), referee assistance (2).

#### 2.4.4 AI and sports industry labor market

The development and dissemination of AI technology has the potential to impact various sectors of the sports industry from the perspective of the labor market (1), notably, but not limited to: (a) coaching and training, (b) scouting and talent identification, (c) sports medicine and injury prevention, (d) content creation, broadcasting and media, (e) CRM, hospitality and marketing (16).

(a) AI has the potential to disrupt the traditional role of coaches and trainers in the sports industry (22). As AI-powered training tools become more advanced (2), some coaches and trainers may need to adapt their skills to incorporate these new technologies into their training regimens. (b) AI algorithms can be used to automate the scouting and talent identification process, potentially reducing the need for human scouts and analysts. This could lead to job displacement in some areas of the industry, particularly for less experienced or entry-level positions. (c) AI can be used to analyze player biometric data and provide real-time feedback to coaches and medical staff. This could lead to more accurate diagnoses and optimized treatment regimens, potentially improving players' health and performance (4). However, it could also lead to a shift in the role of sports medicine professionals, with more emphasis on data analysis and less on traditional medical expertise. (d) AI-powered sports journalism has the potential to automate some aspects of sports reporting (5), such as generating game reports and social media content. This could lead to a reduction in the number of traditional journalism jobs, particularly in areas like data analysis and content generation. (e) AI-powered machine learning algorithms can be used to personalize offerings to individual fans (10), potentially improving the fan experience and increasing revenue for teams and brands (9, 23). However, this could lead to a shift in the type of jobs available in the industry, with more emphasis on data analysis and less on traditional marketing skills.

In brief summary, the development and dissemination of AI technology in the sports industry has the potential to impact various sectors of the labor market in sports industry (1, 4). While there are potential benefits, there are also potential risks and challenges that need to be considered, such as the need for retraining and reskilling, potential job displacement, and the need for appropriate regulations to ensure fair play and ethical use of AI (4).

## 3 Method

### 3.1 Overall methodology

The theoretical framework of technological determinism provides a valuable perspective for exploring the impact of AI on the labor market within the sports industry. The approach focuses on the role of technology as one of driver of workforce changes and ecosystem transformation.

### 3.2 Sampling

Data collection sample has been composed of wide range of international experts in the fields of sports and human resources and/or technologies. The main requirement to interviewees were (1) full time professional experience in sport industry over 5 years; (2) relevant work position in sport industry related to human resources and/or innovation and/or technology transformation. This couple of requirements ensure that interviewees have firsthand experience and knowledge of the impact of AI on the labor market within the sports industry. Due to ethical guidelines, we keep authors identities anonymous in this manuscript. The selection criteria for interviewees are aligned with the research objectives and the theoretical framework. The use of purposive sampling allowed to target experts who are likely to provide valuable insights into the research topic, from interdisciplinary perspective, embracing human resources, AI, sports business. Thus, by selecting interviewees who work in rapidly changing contexts and have a holistic view of the field, the researchers can gather diverse interdisciplinary perspectives and in-depth insights into the impact of AI on the labor market in the sports industry. In the period from March to July 2023 we have contacted over hundred international experts on the LinkedIn and email, and finally managed to collect 14 written interviews only.

### 3.3 Data collection

It refers to the inductive approach in the spirit technological determinism theory. It is based on the literature review and written qualitative, semi-structured interviews (*N* = 14) with sports human resources, management, and technology professionals. We used purposive sampling to target experts who work in the rapidly changing context and have a holistic view of the field. All written interviews were followed by further interrogations, clarifying questions, and precisions. Notably, to improve the accuracy and viability of the interpretation the interpretive validity member check has been done during the data collection process. We have been using paraphrasing and summarization for clarifications to be sure that research participants' viewpoints and thoughts are understood (and interpreted) accurately. Interviewees have spent 50 min on average for answering all our questions. All interviewees gave their written permission to use the information they provided. Interviewees also have been given sight of the summary of this article before giving approval.

The qualitative approach allowed us to gather in-depth insights into the problem and generate new concepts through data synthesis and analysis. Also, considering the transformational and emerging nature of the research topic, this approach and involvement of experts offered an opportunity to capture changes within the labor market in the sports industry. Moreover, taking a qualitative approach to the research was useful since it is not bound by the limitations of quantitative methods and focuses on the primary questions of “how” and “why” the deployment and diffusion of AI technology is transforming the labor in the sports industry and affecting the field ecosystem from different perspectives.

### 3.4 Data analysis

The data analysis process was a rigorous and iterative journey aimed at uncovering rich insights into the impact of AI on the labor market within the sports industry. Building upon the theoretical foundation established through extensive literature review, our analysis unfolded in multiple stages, each contributing to the refinement of our understanding and the development of our findings.

Firstly, we employed an inductive approach to systematize and code the collected data. This initial stage involved identifying key constructs emerging from the data and organizing them into preliminary codes. Through this process, we established a provisional coding frame, which served as the basis for further analysis. Moving into the second stage, we conducted a thorough examination of the materials, engaging in multiple readings to ensure a comprehensive understanding of the data. Utilizing thematic inductive manual coding, we delved into the textual materials, extracting key points and generating detailed codes through a line-by-line analysis. This meticulous approach allowed us to capture nuances within the data and identify merging codes, thus facilitating the establishment of interrelations between key categories and subcategories. These interrelations formed the basis of a taxonomy, which played a crucial role in building a robust theoretical framework for the study. Throughout the analysis process, we embraced reflexivity, continually reflecting on our interpretations and evolving our understanding of the problem at hand. This reflexive stance enabled us to navigate complexities within the data and refine our research direction accordingly.

Finally, we employed a hierarchical coding frame to organize the codes, arranging them based on their relationships to one another. This hierarchical structure provided a coherent framework for synthesizing theoretical and empirical insights, ultimately culminating in the generation of our results. To this end, the data analysis process was characterized by a systematic, iterative, and reflexive approach, resulting in the development of nuanced insights into the impact of AI on the labor market within the sports industry.

## 4 Results and discussion

### 4.1 AI technology, the sports industry's labor market in terms of job demand, and job roles

The development and dissemination of AI technology have had a significant impact on the labor market in the sports industry, leading to changes in job demand and the emergence of new job roles. This impact can be observed in various areas, such as data analytics, fan engagement, sports technology, and performance optimization.

One area that has experienced a substantial increase in job demand is sports data analytics. AI algorithms and machine learning techniques have enabled sports organizations to collect and analyze vast amounts of data, extracting valuable insights for team performance, player scouting, and fan engagement. As a result, there is a growing demand for skilled data analysts who can interpret and derive actionable recommendations from the data.

In addition to data analytics, the integration of AI technology has created new job roles in sports technology. Sports technologists play a vital role in implementing AI-driven solutions, such as motion tracking systems, virtual reality training simulations, and AI-powered coaching tools. These professionals possess a deep understanding of both sports and technology, bridging the gap between technical aspects and the specific needs of the sports industry.

The demand for AI algorithm developers has also increased in the sports industry. These professionals specialize in designing and optimizing algorithms that address specific challenges in sports, such as athlete performance analysis, injury prediction, and game strategy optimization. Their expertise in machine learning, deep learning, and data science is crucial in enabling teams and coaches to make data-driven decisions.

Another area influenced by AI technology in the sports industry is fan engagement. AI-powered tools like chatbots, personalized recommendations, and social media platforms have enhanced the fan experience (10). Professionals specializing in fan engagement leverage AI tools to analyze fan preferences, create targeted marketing campaigns, develop interactive chatbots, and design personalized experiences that keep fans engaged and connected to their favorite teams.

Furthermore, the development of AI technology has led to the emergence of sports performance engineers. These professionals work on developing and optimizing AI-driven tools, such as smart wearables, biomechanical sensors, and tracking devices. They collaborate with sports scientists, engineers, and coaches to improve athlete performance, prevent injuries, and enhance training methodologies using real-time data and AI algorithms.

However, it is worth noting that the adoption and utilization of AI technology in the sports industry may vary across different countries and sports disciplines. The availability of AI-based solutions and their implementation can be influenced by factors such as technological infrastructure, resources, and organizational priorities. In the case of Hungarian ice hockey, for example, AI may not be as widely utilized compared to countries with more advanced implementations. Nonetheless, the potential for AI to penetrate the industry and create new positions related to data analysis and video analysis for professional teams remains a possibility.

The development and dissemination of AI technology have brought about significant changes in the labor market of the sports industry. These changes include increased demand for skilled professionals in areas such as data analytics, sports technology, fan engagement, and sports performance engineering. The integration of AI tools and algorithms has enabled data-driven decision-making, improved fan experiences, and enhanced athlete performance. However, the extent of AI's impact on the labor market may vary depending on the specific country, sport, and level of technological adoption (see limitations and future research direction of current manuscript).

### 4.2 New job opportunities as a result of AI development and dissemination

Based on the data provided, it is evident that the development and dissemination of AI technology in the sports industry have led to the creation of new job opportunities, primarily for highly skilled professionals with expertise in both sports and AI. The data suggests that these new job opportunities are primarily focused on roles such as sports data analysts, sports technologists, AI algorithm developers, fan engagement specialists, AI researchers, AI specialist coaches, and AI sports managers.

The emergence of AI in the sports industry has brought about a shift in the labor market, requiring individuals who possess advanced skills in data analysis, AI algorithms, machine learning techniques, and technological implementation. This demand for highly skilled professionals reflects the industry's increasing reliance on AI-driven tools and analytics to improve various aspects of sports performance, fan engagement, and managerial tasks.

The role of a sports data analyst has become crucial in leveraging AI technology to analyze vast amounts of data and derive actionable insights. These professionals are responsible for interpreting data, identifying patterns, and providing recommendations to enhance team performance, player scouting, and fan engagement. The demand for sports data analysts has increased significantly due to the utilization of advanced AI algorithms and machine learning techniques in data analysis (19).

Sports technologists, on the other hand, play a vital role in implementing AI-driven solutions in sports. Their expertise lies in understanding both the technical aspects of AI and the specific needs of the sports industry. They are responsible for developing and maintaining technologies like motion tracking systems, virtual reality training simulations, and AI-powered coaching tools. The emergence of AI has expanded the scope of their tasks and created new opportunities for professionals with a combination of sports and technological knowledge. AI algorithm developers are in demand as they design and optimize algorithms to address specific challenges in sports. These algorithms support athlete performance analysis, injury prediction, and game strategy optimization. Their expertise in machine learning, deep learning, and data science is critical for developing AI solutions that enhance decision-making processes in the sports industry. Fan engagement specialists are another group of professionals benefiting from the development of AI technology (23). They leverage AI tools to analyze fan preferences, create targeted marketing campaigns, and design personalized experiences. AI has revolutionized fan engagement by enabling interactive chatbots, personalized recommendations, and AI-powered social media platforms. The emergence of AI technology has also impacted academic research in sports science. AI researchers are utilizing AI tools for modeling, data analysis, configuration design, and process organization to advance sports science research. This integration of AI in academic research creates opportunities for specialized professionals with expertise in both AI and sports science. Furthermore, AI specialist coaches have emerged as valuable members of professional athlete coaching teams. They utilize AI-based tools to analyze athlete performance, provide insights for training programs, and optimize game strategies. Their ability to leverage AI algorithms and data analysis contributes to improving athlete performance and overall team success. Lastly, AI sports managers utilize advanced AI technology to optimize managerial tasks in the sports industry. This includes generating contracts with the best data content, analyzing negotiation behavior, and making informed decisions based on AI insights. AI has the potential to streamline and enhance managerial processes, creating job opportunities for professionals who can leverage AI tools effectively.

**Figure 1:**
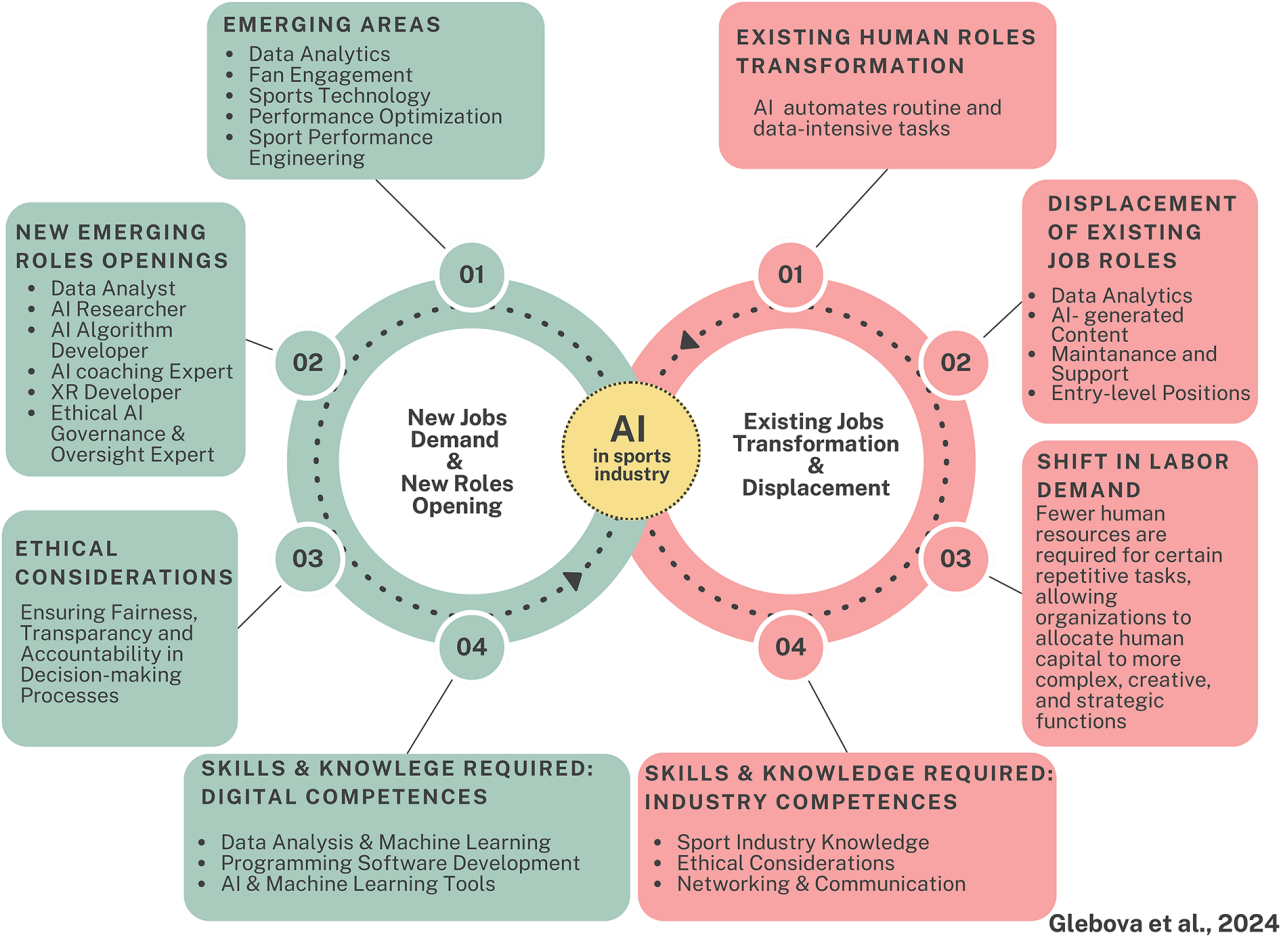
AI dissemination impact on the sports industry labor market.

The dataset analysis shows that the development and dissemination of AI technology in the sports industry have resulted in the creation of new job opportunities. These opportunities primarily exist for highly skilled professionals who possess expertise in both sports and AI. The emergence of roles such as sports data analysts, sports technologists, AI algorithm developers, fan engagement specialists, AI researchers, AI specialist coaches, and AI sports managers showcases the industry's increasing reliance on AI-driven tools and analytics (Figure 1). The demand for these professionals reflects the industry's ongoing integration of AI technology to improve various aspects of sports performance, fan engagement, and managerial tasks.

### 4.3 Skills and knowledge required for the new job roles created by AI technology in the sports industry

The skills and knowledge required for the new job roles created by AI technology in the sports industry can be categorized into two main areas: digital competencies and industry competencies (Figure 1).

*Digital competencies*
  • *Data analysis and machine learning*

The ability to analyze and interpret large volumes of data is crucial in AI-related roles. Professionals should possess strong skills in data preprocessing, statistical analysis, and machine learning techniques. They should be able to apply algorithms and models to extract meaningful insights from sports data (19), enabling data-driven decision-making.

• *Programming and software development*

Proficiency in programming languages, such as Python or R, is essential for implementing AI solutions. Professionals should have a solid understanding of software development principles and practices to design, develop, and deploy AI applications and systems in the sports industry.

• *AI and machine learning tools*

Familiarity with AI and machine learning tools, frameworks, and libraries is necessary. Professionals should be skilled in working with popular tools like TensorFlow, scikit-learn, or PyTorch, enabling them to leverage pre-built models, algorithms, and APIs for sports-related tasks, such as athlete performance analysis or game strategy optimization.

*Industry competencies:*
  • *Sports domain knowledge*

In-depth knowledge of the sports industry is crucial for understanding the context and specific requirements of AI applications (18). Professionals should possess a comprehensive understanding of sports disciplines, game rules, player positions, team dynamics, and performance metrics. This domain knowledge allows them to effectively apply AI techniques to address specific challenges in the sports industry.

• *Ethical considerations*

With the increasing use of AI in sports, professionals in AI-related roles must have a strong understanding of ethical considerations. They should be aware of privacy concerns, bias mitigation, and fairness in AI applications. Ethical decision-making and responsible AI practices are essential to ensure the integrity and trustworthiness of AI systems in the sports industry. By upholding ethical standards and implementing responsible AI protocols, professionals can contribute to fostering an environment of transparency, accountability, and fairness, thereby bolstering public confidence in the ethical deployment of AI technologies in sports.

• *Communication and collaboration*

Effective communication and collaboration skills are essential for professionals working in AI-related roles in the sports industry. They should be able to effectively communicate complex technical concepts to non-technical stakeholders, such as coaches, athletes, or management personnel. Collaboration with multidisciplinary teams, including sports scientists, coaches, and analysts, is crucial to ensure the successful integration of AI technology in sports settings.

The new job roles created by AI technology in the sports industry require a combination of digital competencies, such as data analysis, machine learning, programming, and AI tools, along with industry competencies, including sports domain knowledge, ethical considerations, and effective communication skills. These skills and knowledge are necessary for professionals to harness the potential of AI and drive innovation in the sports industry nowadays.

### 4.4 In what ways has AI technology displaced existing job roles in the sports industry, and what are the consequences of this displacement?

Based on the data provided, it appears that there is a diversity of opinions regarding the displacement of job roles in the sports industry due to AI technology. While some individuals believe that AI has not yet displaced any job roles and instead has introduced new positions, others acknowledge the potential for future displacement (Figure 1).

The possible ways in which AI technology could lead to job displacement in the sports industry are as follows: (1) data analysis, (2) AI-generated content, (3) maintenance and support.

(1) AI algorithms can automate the process of data collection, analysis, and reporting. This may reduce the need for manual data entry and analysis roles, potentially leading to job loss in those areas.
(2) AI technology generates sports-related content, such as match summaries and statistics, which potentially could reduce the demand for traditional sports journalism roles that involve manual content creation.
(3) Advancements in AI may lead to self-maintaining systems and automated technology management, reducing the need for manual intervention in maintenance and support roles.

It is important to note that while job displacement may occur in certain areas, AI technology also brings potential benefits, such as increased efficiency, accuracy, and faster access to information. The consequences of job displacement can vary, including potential job loss for individuals in displaced roles, but also cost savings, improved technology operations, and broader coverage of sports events. It is worth mentioning that specific cases or examples of job displacement in the sports industry are not explicitly provided in the data. However, there are indications of shifts in job roles, where emphasis is placed on working with AI systems as decision support tools rather than direct data manipulation. Thus, while there may not be clear-cut evidence of job displacement in the sports industry due to AI technology, there are potential areas where existing roles could be affected. The consequences of displacement can vary, and it is important to consider the overall impact of AI technology, including both the potential benefits and challenges it brings to the industry.

### 4.5 Trends, the potential future developments of AI in the sports industry, and impact on the labor market

We intended to provide a data-driven analysis of the trends, potential future developments of AI in the sports industry, and their potential impact on the labor market.

According to the study participants, there are following *trends in AI in the sports industry:*

AI-powered performance analytics is becoming increasingly prevalent in the sports industry. Machine learning algorithms can analyze vast amounts of data, including player biometrics, movement patterns, and game statistics, to provide deeper insights into performance, injury prevention, and strategic decision-making.
AI technology is being utilized to enhance fan engagement by delivering personalized experiences (23). AI algorithms analyze fan preferences, behavior, and social media interactions to provide customized content recommendations, targeted marketing campaigns, and interactive chatbots. AI-driven virtual reality XR technologies are being developed to improve training methodologies and provide immersive fan experiences (11). AI algorithms simulate realistic game scenarios, enabling athletes to practice in virtual environments, and provide fans with interactive and immersive viewing experiences. AI is being integrated into sports equipment and wearable devices to enhance athlete performance and safety. AI algorithms analyze sensor data from wearables to optimize equipment design and provide real-time feedback for performance improvement.

#### 4.5.1 Potential future developments of AI in the sports industry

AI algorithms can be further developed to predict performance outcomes and optimize training programs for individual athletes or teams. By analyzing historical performance data and incorporating factors such as player fatigue, injury risk, and environmental conditions, AI can provide more accurate performance predictions and personalized training recommendations. AI has a potential to provide real-time decision support for coaches during games. These systems can analyze ongoing game data, player performance metrics, and opponent strategies to suggest optimal game plans, substitutions, and tactical adjustments (24). As mentioned above, AI algorithms are trained to generate sports-related content, such as automated game summaries, match reports, and social media posts. This can streamline content creation processes and provide real-time updates to engage fans more effectively.

#### 4.5.2 Impact on the labor market

The advancements and integration of AI in the sports industry may have several impacts on the labor market:

a. *New job roles*

The development and implementation of AI technologies will likely create new job roles requiring expertise in AI, data analysis, and sports domain knowledge. These roles may include sports data analysts, AI algorithm developers, sports technologists, XR developers, and AI marketing experts.

b. *Job transformation*

Existing job roles may undergo transformation as AI automates routine and data-intensive tasks. Professionals in roles such as data entry, manual data analysis, and content creation may need to upskill or transition to new roles that require a deeper understanding of AI technologies and their integration into the sports industry.

c. *Efficiency and productivity*

AI-powered automation can enhance efficiency and productivity in various areas of the sports industry, furthermore, it may completely change industry paradigms, looking evident nowadays, such as stadium attendance, home advantage, and current business models (25). This may lead to a shift in labor demand, where fewer human resources are required for certain repetitive tasks, allowing organizations to allocate human capital to more complex, creative, and strategic functions.

d. *Ethical considerations*

The adoption of AI in the sports industry raises ethical considerations, such as ensuring fairness, transparency, and accountability in decision-making processes. This may necessitate the creation of new roles or responsibilities related to ethical AI governance and oversight.

The impact on the labor market always depends on various factors, including the pace of AI adoption, industry regulations, and the ability of individuals to adapt and acquire the necessary AI-related skills. Organizations and individuals need to proactively embrace these changes, fostering a culture of lifelong learning and skill development to stay competitive in an AI-driven sports industry.

## 5 Conclusion

The application of AI in the sports industry is experiencing significant growth, with several prominent trends and potential future developments shaping its trajectory. Advanced performance analytics, personalized fan engagement, virtual reality and augmented reality experiences, and AI-powered sports equipment and wearables are among the key trends observed in the industry. These developments offer vast potential to enhance athlete performance, fan experiences, and overall operational efficiency for all stakeholders in the industry, with a high probability for restructuring of entire sports ecosystem. Subsequently, it affects and will further affect the labor market.

Looking ahead, potential future developments in AI within the sports industry include enhanced performance prediction, real-time decision support systems for coaches, and AI-generated content creation. These advancements hold the promise of delivering more accurate performance predictions, optimizing training programs, assisting coaches in making real-time strategic decisions, and automating content creation processes. The labor market implications of AI in the sports industry are multifaceted. On one hand, new job roles are likely to emerge, demanding a blend of AI expertise, data analysis skills, and sports domain knowledge. Professionals in roles such as sports data analysts, AI algorithm developers, sports technologists, XR developers, and AI marketing experts may find increasing opportunities. On the other hand, certain job roles may undergo transformation as AI automates routine and data-intensive tasks. This transformation could require individuals to upskill or transition to roles that require a deeper understanding of AI technologies.

Furthermore, the integration of AI in the sports industry raises ethical considerations, including the need for fairness, transparency, and accountability in decision-making processes. This may necessitate the creation of new roles or responsibilities focused on ethical AI governance and oversight.

The adoption of AI in the sports industry has the potential to revolutionize various aspects of the field. While it offers opportunities for performance optimization, personalized fan experiences, and operational efficiency, it also presents challenges in terms of job displacement and ethical implications. Organizations and individuals must proactively embrace these changes, fostering a culture of continuous learning and adaptation to navigate the evolving landscape of an AI-driven sports industry. By doing so, they can maximize the benefits of AI while addressing potential concerns and ensuring a sustainable and inclusive future for the sports industry as a whole.

While the discussion on AI in the sports industry provides valuable insights, there are certain limitations that should be acknowledged. (1) First, the analysis is based on a conversation and exchange of information within the context of a single chat session. The information provided may not capture the full scope of research and developments in the field. A more comprehensive and systematic analysis of existing literature, case studies, and industry reports would be necessary to obtain a comprehensive understanding of the topic. (2) Second, the analysis relies heavily on subjective opinions and perspectives expressed during the written interview process. While these viewpoints provide valuable insights, they may not represent a complete consensus within the industry. Conducting surveys, interviews, or expert panels could provide a more comprehensive understanding of the current landscape and future trends in AI adoption within the sports industry. (3) Third, the available data and research on the labor market impact of AI in the sports industry are limited. Further research is needed to examine the extent to which AI technologies have already displaced existing job roles and the implications for workforce dynamics. Longitudinal studies tracking job market trends and qualitative investigations into the experiences of individuals affected by AI-driven changes in the sports industry would provide valuable insights. (4) Complementing the only qualitative insights obtained in the current study, which is a limitation by itself, future research could employ quantitative methodologies to assess the quantitative impact of AI on the sports labor market. Statistical analyses could be used to quantify changes in work formats, employment patterns, job satisfaction levels, and skill requirements resulting from AI adoption in the sports industry.

Future research directions can build upon the existing analysis and address these limitations. Studies can focus on conducting in-depth literature reviews to explore the latest advancements in AI technologies specific to the sports industry. Additionally, quantitative research can be conducted to analyze the labor market impact of AI adoption, examining factors such as job displacement, skill requirements, and the emergence of new job roles. Ethical considerations, including fairness, transparency, and accountability in AI applications within the sports industry, warrant further investigation (26). To broaden the scope of inquiry, future research could conduct comparative studies across different sports sectors (e.g., professional leagues, grassroots organizations, sports technology companies) and geographical zones to examine variations in AI adoption and its impact on the labor market. Comparative analyses could shed light on sector-specific and demographical challenges and opportunities related to AI integration in sports.

Furthermore, interdisciplinary research that combines expertise from sports science, computer science, data analytics, and ethics would contribute to a more comprehensive understanding of the challenges and opportunities posed by AI in sports (27). Long-term studies tracking the evolution of AI technologies and their impact on various stakeholders in the industry would provide valuable insights into the sustainability and long-term effects of AI adoption.

Future research projects should aim to address the limitations of this study, expand the depth and breadth of analysis, and explore new dimensions of AI adoption in the sports industry, facilitating evidence-based decision-making and informing industry practices.

## Data Availability

The data supporting the conclusions of this article is available upon reasonable request.
